# Exercise Profile and Diastolic Functions Measured via Tissue Doppler Imaging of Fibromyalgia Patients

**DOI:** 10.14740/jocmr1799w

**Published:** 2014-03-31

**Authors:** Ozlem Balbaloglu, Huseyin Ede, Sadiye Yolcu, Hakan Ak, Nermin Tanik, Gulacan Tekin

**Affiliations:** a Department of Physical Medicine and Rehabilitation, Faculty of Medicine, Bozok University, Yozgat, Turkey; b Department of Cardiology, Faculty of Medicine, Bozok University, Yozgat, Turkey; c Department of Emergency Medicine, Faculty of Medicine, Bozok University, Yozgat, Turkey; d Department of Neurosurgery, Faculty of Medicine, Bozok University, Yozgat, Turkey; e Department of Neurology, Faculty of Medicine, Bozok University, Yozgat, Turkey

**Keywords:** Fibromyalgia, Diastolic function, Electrocardiography, Echocardiography, Exercise treadmill test

## Abstract

**Background:**

Our aim was to evaluate electrocardiographic and echocardiographic properties and exercise response of patients with fibromyalgia (FM).

**Methods:**

The study included 60 women with primary FM and 30 healthy individuals. Resting electrocardiography, echocardiography and exercise treadmill test were used to compare these two groups. At apical four-chamber window, samples of transmitral diastolic inflow and tissue Doppler imaging of left ventricle lateral wall were obtained. Left ventricle ejection fraction was measured via modified Simpson’s method. Exercise duration, maximal exercise capacity, maximal heart rate (HR) (bpm), maximal HR (%), rate-pressure product at maximal HR (bpm × mmHg), heart rate recovery 1 (bpm), heart rate recovery 2 (bpm) and chronotropic reserve (%) values were calculated.

**Results:**

Resting HR and QTc values were similar in both groups. Echocardiographic measurements in both groups did not reveal statistically significant difference except left ventricle end-diastolic diameter and left atrial diameter. Parameters related to diastolic function of the left ventricle did not differ significantly in both groups. Also, there was not any significant difference between the groups for E/E’ ratio and chronotropic reserve. Exercise treadmill test results were statistically similar for both groups.

**Conclusion:**

Patients with FM presented a normal HR response to exercise and those patients had normal diastolic function similar to their healthy controls.

## Introduction

Fibromyalgia (FM) is known as a chronic syndrome characterized by widespread pain and discomfort [1]. In addition to widespread pain present for more than 3 months, patients with FM often report fatigue, nonrestorative sleep, dysfunction mood disturbance, stiffness and cognitive complaints [2-5]. Widespread pain is described as pain above and below the waist and on both sides of the body. Additionally, axial skeletal pain (in the cervical spine, anterior chest, thoracic spine, or lower back) must be seen. According to American College of Rheumatology, a patient must have pain on digital palpation at 11 of 18 predesignated sites, commonly described as tender points, for diagnosis of FM [6]. While the etiology of FM is not fully understood, the data have suggested that FM may result from dysfunction of the autonomic nervous system. This dysfunction has been reported at rest and after a physiological stress such as exercise [7]. However, few studies have searched such relation. Changes in autonomic function have been suggested to increase the risk for cardiovascular events and mortality [8]. In this regard, heart rate (HR) response to a graded exercise test has been largely used for assessing risk and prognosis in patients with overt or subclinical cardiovascular diseases [9, 10]. Similarly QTc and HR variability are other parameters used for assessing autonomic dysfunction at rest. Diastolic dysfuntion is also related to cardiovascular mortality and mortality [11]. There is limited knowledge in the literature about diastolic dysfuntion in FM patients.

In this study, we aimed to compare cardiovascular properties of the patients with FM to those of normal population by using electrocardiographic, echocardiographic and exercise treadmill test findings.

## Materials and Methods

Sixty female patients diagnosed with FM by Physical Medicine and Rehabilitation Department according to American Collage of Rheumatology criteria and with chest pain at the same time were included in the study between May 2013 and January 2014. All patients were evaluated by Cardiology Department. Resting electrocardiography (ECG), echocardiography and treadmill exercise tests were carried out for all the patients. The results were compared to those of the control group. The study was approved by the ethical committee of our institution and informed consent was obtained from each patient.

Exclusion criteria were: 1) cardiovascular involvement such as arrhythmias, arterial hypertension, heart failure, conduction disturbances, myocarditis and pericarditis; 2) tobacco usage; 3) use of chronotropic and antihypertensive drugs; 4) other chronic rheumatologic diseases such as systemic lupus erythematosus and rheumatoid arthritis; 5) diabetes mellitus; 6) dyslipidemia and 7) chronic kidney disease. At entry, patients and control subjects were not taking any kind of medication.

### Resting ECG

Resting ECG of all patients and controls was obtained after the rest of 5 min in the room. Of this ECG, resting HR and QTc interval were measured by using the Bazett formula.

### Treadmill exercise test

Modified Bruce treadmill protocol was used to assess the cardiac autonomic modulation in patients with FM in response to exercise. The test was stopped due to either patient’s demand or reaching maximal target HR. HR was continuously recorded at rest, during exercise and at recovery, using a 12-lead electrocardiogram. HR response during exercise was evaluated by the chronotropic reserve measured with the formula (chronotropic reserve = (peak HR - resting HR/220 - age - resting HR) × 100). Chronotropic incompetence was determined when subjects failed to achieve more than 80% of the chronotropic reserve. The recovery period was set at 2 min using the initial workload (1.9 mph). Heart rate recovery (HRR) was defined as the difference between HR at peak exercise and at both first (HRR1) and second (HRR2) minutes after exercise. Maximal HR in percent of target HR, rate-pressure product (HR multiplied with blood pressure at that moment), maximal exercise capacity in the standard metabolic equivalent (MET) and exercise duration were used for analysis.

### Echocardiographic examination

In echocardiographic examination, left ventricle end-diastolic diameter (LVEDD), left ventricle end-systolic diameter, posterior wall diastolic thickness, interventricular septum diastolic thickness (IVSd), left atrial size, aortic diameter, left ventricle ejection fractions (LVEF) by using modified Simpson’s method and pulmonary arterial systolic pressure (PASP) were measured in accordance with the rules set by American Echocardiography Society [12]. At apical four-chamber window, by using pulse wave Doppler, mitral E and A waves were measured. E/A ratios of all patients were calculated. All measurements were carried out at the end of expirium. By using pulse wave tissue Doppler imaging, all diastolic myocardial velocities were obtained. In this study, to make the evalution more practical, sample volume at left ventricle lateral wall basal segment was used to obtain E’ and A’ wave velocities at apical four-chamber window. E/E’ ratios were calculated accordingly. All Doppler measurements were carried out for five subsequent cycles and averages of all five measurements were used for statistical analysis.

### Statistical analysis

Statistical analysis was carried out by using SPSS 11.5 (Statistical Package for Socia Sciences-SPSS, Inc., Chicago, IL, USA) programme. While categorized variables were expressed as percentage, continuous variables were expressed as arithmetic average ± standard deviation. One-sample Kolmogorov-Smirnov test was applied to check whether continuous variables are distributed normally. All continuous variables of the study were normally distributed. Possible associations between continuous variables were analyzed by using Pearson’s correlation test. Possible statistical difference between group characteristics was analyzed by using Student’s t test. P value < 0.05 was considered statistically significant.

## Results

Sixty women with primary FM (age: 37.4 ± 7.2 years; BMI: 28.9 ± 4.9 kg/m^2^) and 30 healthy individuals (age: 39.1 ± 8.5 years; BMI: 30.2 ± 5.3 kg/m^2^) were included in the study.

Demographic properties of the patients with FM and the control group (CTRL) are shown in Table 1. Both groups were statistically similar in respect to age, weight, height and BMI.

**Table 1:** Demographic Properties of the Patients With Fibromyalgia (FM) and the Control Group

	Control (n = 30)	FM (n = 60)	P
Age (year)	39.1 ± 8.5	37.4 ± 7.2	0.565
Weight (kg)	76 ± 13	73 ± 13	0.581
Height (cm)	158 ± 5	159 ± 8	0.798
BMI (kg/m^2^)	30.2 ± 5.3	28.9 ± 4.9	0.478

Resting HR and QTc values were similar statistically in both groups (P = 0.315 and P = 0.389). Except LVEDD (FM: 44 ± 3 mm vs. CTRL: 46 ± 1 mm, P < 0.05) and LA diameter (FM: 31 ± 5 mm vs. CTRL: 35 ± 2 mm, P < 0.05), all other echocardiographic measurements (LVEF, aort diameter, PASP and IVSd) of both groups were not significantly different. In both groups, parameters related to diastolic function of the left ventricle measured via standard method (mitral flow E/A ratio, P = 0.311) or tissue Doppler imaging (left lateral basal segment E’/A’ ratio, P = 0.917) did not differ significantly. Additionaly, there was not any significant difference between the groups for E/E’ ratio (P = 0.66). The results of electrocardiographic and echocardiographic examinations are introduced in Table 2.

**Table 2:** Electrocardiographic and Echocardiographic Results of the Patients With Fibromyalgia (FM) and the Control Group

	Control (n = 30)	FM (n = 60)	P value
QTc (ms)	404 ± 12	408 ± 14	0.389
Resting heart rate (bpm)	77 ± 10	73 ± 11	0.315
LVEF (%)	65 ± 2	66 ± 2	0.129
LA diameter (mm)	35 ± 2	31 ± 5	< 0.05
Aort diameter (mm)	25 ± 6	26 ± 7	0.886
LVEDD (mm)	46 ± 1	44 ± 3	< 0.05
IVSd (mm)	8.7 ± 0.8	8.1 ± 0.9	0.066
PASP (mmHg)	22.7 ± 3.2	22.7 ± 3.1	1.000
Mitral flow E/A	1.6 ± 0.6	1.5 ± 0.3	0.311
TDI left lateral basal segment E’/A’	1.72 ± 0.65	1.70 ± 0.55	0.917
E/E’	5.8 ± 1.2	6.1 ± 1.6	0.660

We did not find any significant correlation for reached maximal HR between FM and the control group (166 ± 11 bpm and 165 ± 9 bpm respectively, P = 0.725). Also rate-pressure product at maximal HR was not siginificantly different in CTRL group compared to FM group (CTRL: 26,542 ± 4,316 bpm × mmHg, FM: 24,163 ± 4,133 bpm × mmHg, P = 0.134). Exercise duration and maximal exercise capacity in MET were similar statistically for both groups (FM: 555 ± 98 sec vs. CTRL: 488 ± 104 sec, P = 0.079 and FM: 9.2 ± 2.2 MET vs. CTRL: 8.3 ± 2.3 MET, P = 0.289). The groups did not differ significantly in respect to chrontropic reserve (FM: 80 ± 12% vs. CTRL: 78 ± 9%, P = 0.620) (Table 3).

**Table 3:** Exercise Test Results of the Patients With Fibromyalgia (FM) and the Control Group

	Control (n = 30)	FM (n = 60)	P value
Exercise duration (sec)	488 ± 104	555 ± 98	0.079
Maximal exercise capacity (in MET)	8.3 ± 2.3	9.2 ± 2.2	0.289
Maximal heart rate (bpm)	165 ± 9	166 ± 11	0.725
Maximal heart rate (%)	92 ± 4	92 ± 5	0.809
Rate-pressure product at maximal heart rate (bpm × mmHg)	26,542 ± 4,316	24,163 ± 4,133	0.134
Heart rate recovery 1 (bpm)	36 ± 12	39 ± 12	0.438
Heart rate recovery 2 (bpm)	58 ± 13	62 ± 11	0.372
Chronotropic reserve (%)	78 ± 9	80 ± 12	0.620

HRR values of both groups were similar at the end of first and second minute (Table 3). All subjects enrolled for the sudy had normal HRR1 (value of > 12 bpm) and had normal HRR2 (value of > 24 bpm).

## Discussion

We aimed to compare cardiovascular properties of the patients with FM to those of normal population by using electrocardiographic, echocardiographic and exercise treadmill test findings.

We found that patients with FM showed similar HR response during and after exercise as volunteer healthy subjects did. We also found that the patients with FM had similar chronotropic reserve as their control group. It has been reported that the patients with FM have autonomic dysfunction which is one of the suspected reason for increased cardiovascular mortality and morbidity [13].

Autonomic dysfunction may be well assessed by evaluating the resting ECG parameters such as QTc interval and HR variability. In this study, we used corrected QT interval. HR-corrected QT interval duration (QTc) has been shown to be related with cardiac autonomic dysfunction in patients with systemic lupus erythematosus, rheumatoid arthritis and chronic inflammatory arthritis [14-16]. In the literature, there is only one study evaluating FM patients [17]. In that study, average corrected QT of patients with FM was significantly longer than patients with chronic fatigue syndrome (417 ± 25 ms vs. 372 ± 22 with P < 0.0001). In our study, we compared corrected QT interval of FM patients with healthy individuals (408 ± 14 ms vs. 404 ± 12 with P = 0.389 respectively). If QTc interval of patients with FM in our study was compared to that of patients with FM in the study by Naschitz et al [17], it would be clearly seen that corrected QT intervals of both FM groups were within normal range defined for general female population, accepted as less than or equal to 470 ms [18]. So it cannot be said easily that the patients with FM had prolonged QTc interval while all the values in concern were within normal range. In this report, the study tried to explain this increased mortality solely via autonomic dysfunction but they never mentioned about any arrhythmic events which are expected to be direct result of autonomic dysfunction [17]. To prove the presence of such relation, long-term follow-up studies should be designed.

Echocardiography is one of the most helpful and frequently used tool in daily cardiology practice to evaluate systolic and diastolic functions of the heart [19]. Diastolic dysfunction, measured by standard mitral flow pattern or tissue Doppler imaging, is a well-known predictor of cardiovascular events and is one of the firstly impaired functions of the heart in concern [20]. In the literature, there is no study evaluating the correlation between autonomic dysfunction and diastolic dysfunction in patients with FM. We did not find any statistically significant difference between FM and control group for diastolic functions. Both groups had similar mitral flow pattern, pulse wave E/A ratio (1.5 ± 0.3 for FM vs. 1.6 ± 0.6 for CTRL, P = 0.311) and left lateral wall basal segment pulse wave TDI values, E’/A’ ratio (1.70 ± 0.55 for FM vs. 1.72 ± 0.65 for CTRL, P = 0.917) and also E/E’ values, very specific parameter for left ventricle filling pressure which is expressed via pulmonary capillary wedge pressure were similar for both groups (6.1 ± 1.6 for FM vs. 5.8 ± 1.2 for CTRL, P = 0.660). E/E’ values of > 12 are accepted as abnormal and none of our patients with FM and control groups had E/E’ value of > 12. In other words, both groups had similar diastolic properties without increased pulmonary capillary wedge pressure.

Exercise ECG test is a very useful method in assessing the cardiac status [21]. Exercise duration, exercise capacity measured in MET, maximal HR expressed as a percentage of target HR, chronotropic reserve and rate-pressure product at maximal HR are some of the parameters used to define the cardiovascular risk of individuals [21]. In our study, all mentioned parameters were similar in both of two groups. Bardal et al [22] reported that patients with FM have similar metabolic and cardiovascular responses to submaximal exercise as in healthy people. Our results were parallel to the findings of Bardal et al [22]. Additionally, we used rate-pressure product to evaluate hemodynamic response to exercise. In this respect, this study was the first study in the literature using the rate-pressure product for comparison of patients with FM and normal population. In our study, we found that average rate-pressure product of FM group was statistically similar to that of control group (24,163 ± 4,133 bpm × mmHg for FM vs. 26,542 ± 4,316 bpm × mmHg for CTRL, P = 0.134). As an index of myocardial oxygen consumption of myocardium, rate-pressure products of both groups were within intermediate range. The importance of such finding needs further investigation.

It is known that markers reflective of autonomic nervous system function can predict major cardiovascular events, total mortality and sudden cardiac death [21]. HRR is one of these markers and decreased HRR, which is HRR1 < 12 bpm and HRR2 < 24 bpm, has been associated with death and cardiac events in a number of populations [21]. In our study, HRR1 (39 ± 12 bpm for FM vs. 36 ± 12 bpm for CTRL, P = 0.438) and HRR2 values (62 ± 11 bpm for FM vs. 58 ± 13 bpm for CTRL, P = 0.372) were similar. In the literature, it had been reported that patients with FM had significantly lower values compared to normal population [13]. When this article was examined in detail, it would be found that all subjects of both groups had clinically normal HRR1 (24.5 ± 3 bpm vs. CTRL: 32.6 ± 2 bpm, P = 0.059) and HRR2 (34.3 ± 4 bpm vs. CTRL: 50.8 ± 3 bpm, P = 0.002) [13]. So comparing categorically two normal populations could not give clinically important difference, although there was statistically difference. So our results did not oppose the literature rather supported.

The other marker reflecting autonomic dysfunction is the chronotropic incompetence, defined as failure of the HR to achieve above 80% of the chronotropic reserve during exercise, independent predictor of all-cause mortality in healthy and diseased subjects [13, 23]. Chronotropic reserves of both groups were similar in our study (80 ± 12 for FM vs. 78 ± 9 for CTRL, P = 0.620). In the literature, there have been few studies searching relation between FM and chronotropic reserve [13]. While Ribeiro et al found average chronotropic reserve value FM when compared with CTRL (72.5 ± 5 vs. 106.1 ± 6, respectively) [13]. Similarly 46.7% of our FM group had CI, while it was 57.1% in the study by Ribeiro et al [13]. In both studies, all factors which could affect chronotropic reserve were excluded, so the difference between the average values of both studies may be due to different treadmill protocols used in the studies (modified Balke vs. modified Bruce) and this needs further investigation.

### Conclusions

Patients with FM presented a normal HR response to exercise, suggesting normal cardiac autonomic function. Also we found that patients with FM had normal diastolic function compared to control group. All these findings showed normal cardiac properties rather than properties related to increased risk for cardiac events and mortality. But large scale prospective studies should be designed to clarify in this field.
